# Prodifferentiation Activity of Novel Vitamin D_2_ Analogs PRI-1916 and PRI-1917 and Their Combinations with a Plant Polyphenol in Acute Myeloid Leukemia Cells

**DOI:** 10.3390/ijms17071068

**Published:** 2016-07-05

**Authors:** Matan Nachliely, Ehud Sharony, Narasimha Rao Bolla, Andrzej Kutner, Michael Danilenko

**Affiliations:** 1 Department of Clinical Biochemistry and Pharmacology, Ben Gurion University of the Negev, Beer Sheva 841051, Israel; matanna@post.bgu.ac.il (M.N.); ehudshar@gmail.com (E.S.); 2 Department of Chemistry and Department of Pharmacology, Pharmaceutical Research Institute, Warsaw 01-793, Poland; n.bolla@ifarm.eu (N.R.B.); a.kutner@ifarm.eu (A.K.)

**Keywords:** acute myeloid leukemia, analogs of 1α,25-dihydroxyvitamin D_2_, vitamin D receptor, retinoid X receptor, carnosic acid, cell differentiation, cell cycle distribution

## Abstract

1α,25-dihydroxyvitamin D_3_ (1,25D3) is a powerful differentiation inducer for acute myeloid leukemia (AML) cells. However, 1,25D3 doses required for differentiation of AML cells may cause lethal hypercalcemia in vivo. There is evidence that vitamin D_2_ is less toxic than vitamin D_3_ in animals. Here, we determined the differentiation effects of novel analogs of 1α,25-dihydroxyvitamin D_2_ (1,25D2), PRI-1916 and PRI-1917, in which the extended side chains of their previously reported precursors (PRI-1906 and PRI-1907, respectively) underwent further 24*Z* (24-*cis*) modification. Using four human AML cell lines representing different stages of myeloid maturation (KG-1a, HL60, U937, and MOLM-13), we found that the potency of PRI-1916 was slightly higher or equal to that of PRI-1906 while PRI-1917 was significantly less potent than PRI-1907. We also demonstrated that 1,25D2 was a less effective differentiation agent than 1,25D3 in these cell lines. Irrespective of their differentiation potency, all the vitamin D_2_ derivatives tested were less potent than 1,25D3 in transactivating the DR3-type vitamin D response elements. However, similar to 1,25D3, both 1,25D2 and its analogs could strongly cooperate with the plant polyphenol carnosic acid in inducing cell differentiation and inhibition of G1–S cell cycle transition. These results indicate that the 24*Z* modification has contrasting effects on the differentiation ability of PRI-1906 and PRI-1907 and that the addition of a plant polyphenol could result in a similar extent of cell differentiation induced by different vitamin D compounds. The enhanced antileukemic effects of the tested combinations may constitute the basis for the development of novel approaches for differentiation therapy of AML.

## 1. Introduction

Acute myeloid leukemia (AML) remains a devastating disease and is characterized by the disruption of the differentiation program in early myeloid progenitors. AML treatment is based on cytotoxic combination chemotherapy, which has changed little in the last four decades. While 50%–70% of younger patients initially respond to remission-induction therapy, incurable relapses are inevitable for most patients. Standard chemotherapy is mostly unsuitable for the elderly individuals representing the majority of patients with AML [1,2,3].

**Figure 1:**
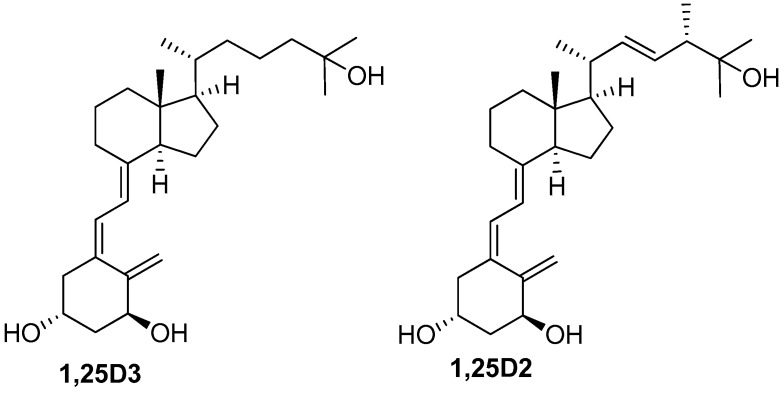
Structures of 1α,25-dihydroxyvitamin D_3_ (1,25D3) and 1α,25-dihydroxyvitamin D_2_ (1,25D2).

The hormonal form of vitamin D_3_ (cholecalciferol), 1α,25-dihydroxyvitamin D_3_ (1,25D3; Figure 1), is one of the most powerful anticancer agents, which can induce differentiation, growth arrest, apoptosis, or a combination of these in various types of cancer cells [4,5]. Furthermore, low levels of the circulating 1,25D3 precursor 25-hydroxyvitamin D_3_ (25D3) are associated with shorter survival, worse prognosis, and adverse treatment outcome in myeloid leukemias and other types of cancer [6,7,8,9]. In contrast to conventional or targeted chemotherapeutic drugs, 1,25D3 has only one major type of in vivo toxicity that can be life threatening at pharmacologically effective doses: hypercalcemia. Although this precludes it from use in clinical oncology, the potential benefits from 1,25D3-based anticancer therapy may be substantial. Therefore, a great number of synthetic 1,25D3 analogs (>3000) have been synthesized to date aiming to obtain a potent drug with reduced calcemic toxicity. However, apart from some encouraging results, the currently existent compounds that have reached clinical trials have not yet shown consistent clinical responses, and hypercalcemia still remains a major limiting factor [10,11,12]. One solution to this challenge is the use of compounds that can potentiate the anticancer effects, and not the toxicity, of low concentrations of vitamin D derivatives (VDDs) [13,14,15,16,17]. For instance, we and others have shown that plant polyphenols, e.g., carnosic acid (CA) from rosemary and silibinin from milk thistle, markedly potentiate the differentiation-inducing effects of low concentrations of VDDs on AML cell lines and patient-derived leukemic blasts [17,18,19,20,21,22,23].

**Figure 2:**
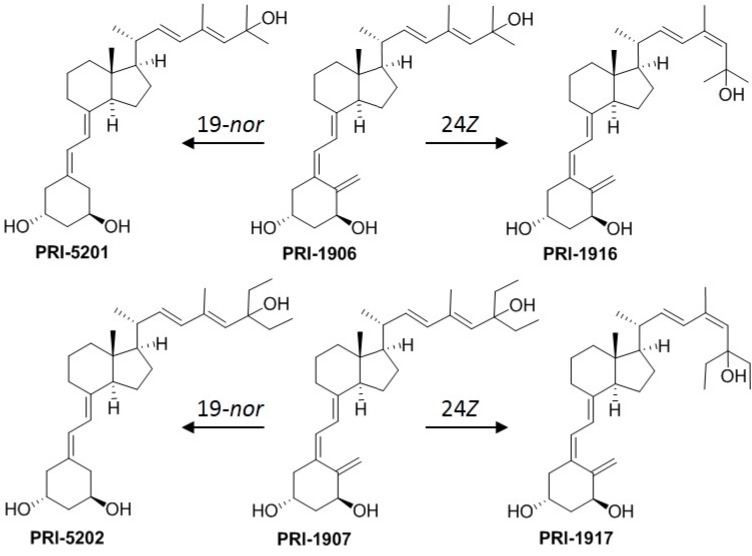
Structural 19-*nor* and 24*Z* modifications of PRI-1906 and PRI-1907, the side-chain homologated and conjugated analogs of 1α,25-dihydroxyvitamin D_2_.

Vitamin D_2_ (ergocalciferol) has been shown to exhibit essentially the same general biological effects as vitamin D_3_, though there is some controversy regarding the relative biopotency of the two compounds (reviewed in [24]). Importantly, there is evidence that vitamin D_2_ and its derivatives are less toxic than vitamin D_3_ compounds in animals [24,25,26]. In the previous study [17] we characterized the in vitro antileukemic effects of PRI-5201 and PRI-5202 (Figure 2), the double point-modified analogs of 1,25-dihydroxyvitamin D_2_ (1,25D2, Figure 1). These analogs have the optimized length and unsaturation of the side-chain of their direct precursors (PRI-1906 and PRI-1907 [27], respectively) with the addition of a very effective 19-*nor* modification (Figure 2) [28]. The results demonstrated superior differentiation-inducing effects of PRI-5201 and PRI-5202, as compared to 1,25D3, PRI-1906, and PRI-1907, in HL60, U937 and MOLM-13 human leukemia cell lines [17]. In an attempt to further optimize the side-chains of PRI-1906 and PRI-1907, we here introduce a 24*Z* (24-*cis*) modification to the side-chain conjugated diene system resulting in two novel analogs, PRI-1916 and PRI-1917 [29] (see Figure 2).

In the present study, we compared the prodifferentiation activity of the novel geometric 24*Z* isomers to that of PRI-1906 and PRI-1907, 1,25D2 and 1,25D3, using a panel of four human AML cell lines representing different stages of myeloid maturation (KG-1a, HL60, U937, and MOLM-13). The ability of the new 24*Z* analogs of 1,25D2 to cooperate with CA was also examined in this study. Furthermore, a comprehensive activity comparison between the parent vitamin D compounds (1,25D3 and 1,25D2) was conducted in all the experimental models tested here.

## 2. Results

### 2.1. Comparison of the Differentiation-Inducing Effects of Different Vitamin D Derivatives in a Panel of AML Cell Lines

**Figure 3:**
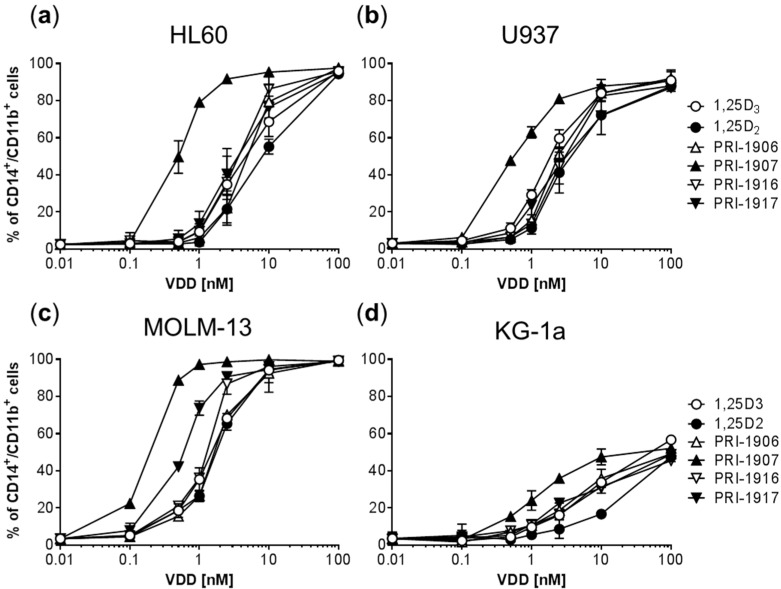
Comparison of the differentiation-inducing effects of different vitamin D derivatives on AML cells. (**a**–**d**) Cells were incubated with the indicated agents or vehicle (≤0.2% ethanol) for 96 h. The expression of CD14 and CD11b was determined by flow cytometry. The percentage of the double-positive CD14^+^/CD11b^+^ cell population is presented. The data are the means ± SD of at least four independent experiments.

Using a flow cytometric cell differentiation assay in four human AML cell lines, we first determined the dose-response relationship of the novel 1,25D2 analogs, PRI-1916 and PRI-1917, in comparison with their direct precursors (PRI-1906 and PRI-1907, respectively) and the parent compounds, 1,25D2 and 1,25D3. The AML cell lines used represented different stages of myeloid differentiation: KG-1a (leukemia stem-like cells), HL60 (myeloblastic leukemia), U937 (myelomonocytic leukemia), and MOLM-13 (monocytic leukemia). The extent of myeloid differentiation was assessed by bivariate analysis of surface expression of the specific monocytic marker CD14 and the general myeloid marker CD11b. Treatment with the vitamin D compounds for 96 h resulted in a dose-dependent increase in the levels of each marker (data not shown) as well as in the percentage CD14^+^/CD11b^+^ double-positive cells (Figure 3) in all the cell lines tested.

The results demonstrated that, similar to the previously reported data [17,30], PRI-1907 exhibited much higher differentiation potency than PRI-1906 in all the employed AML cell lines (Figure 3 and Table 1). However, the impacts of the new 24*Z* modification on the activities of PRI-1906 and PRI-1907 were strikingly different. Thus, the potency of PRI-1916 was slightly higher than or equal to that of PRI-1906, which contains natural alkyl branches at C-25. On the other hand, the potency of PRI-1917 in the four cell lines was found to be consistently lower, as compared to PRI-1907 containing homologated chains at C-25 (Figure 3 and Table 1). Interestingly, the MOLM-13 cell line, which has been reported to express wild-type p53 protein [31], exhibited higher sensitivity to all the VDDs tested compared to the cell lines which, according to the previous studies, lack the expression of p53 mRNA: HL60 [32], U937, and KG-1 [33]. In addition, we found that 1,25D2 was significantly less potent as the differentiation inducer than 1,25D3 in all the four cell lines (Figure 3 and Table 1).

**Figure 4:**
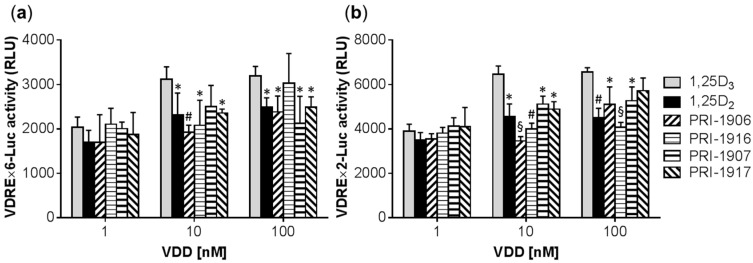
Vitamin D_2_ derivatives are less effective than 1,25D3 in transactivating vitamin D response element (VDRE). U937 cells were transiently co-transfected with VDRE×6-Luc (**a**) or VDRE×2-Luc (**b**) and Renilla luciferase constructs, and incubated with the indicated agents for 24 h. The relative firefly luciferase activity (means ± SD) was calculated from the data of 3 individual experiments performed in quadruplicate. The basal VDRE activity, in the absence of vitamin D derivatives (VDDs), was negligible (50–100 RLU) for both VDRE reporter constructs. *, *p* < 0.05; ^#^, *p* < 0.01; and ^§^, *p* < 0.001 vs. 1,25D3 at each concentration of the VDDs.

To determine whether the observed differences between the differentiation potencies of the employed VDDs correlate with their ability to transactivate the vitamin D response element (VDRE), we performed the luciferase reporter gene assay in U937 cells using two different VDRE reporter constructs: VDRE×6-Luc containing six copies of a direct repeat 3 (DR3)-type VDRE consensus sequence [34] and VDRE×2-Luc containing two copies of the osteopontin (Spp1) DR3-type VDRE sequence [4]. As shown in Figure 4, the two different reporter systems exhibited very similar transactivation patterns in response to the VDDs. Particularly, at a relatively low concentration of the VDDs (1 nM), there were no significant differences between the effects of these compounds, whereas, at higher concentrations (10–100 nM), all of the vitamin D_2_ derivatives generally displayed lower efficiency in transactivating VDRE compared to 1,25D3 without consistent differences among the individual agents (Figure 4). This supports our previous findings showing that even the strongest 1,25D2-related differentiation inducers, such as PRI-5202 and PRI-1907, were less effective DR3-VDRE activators than 1,25D3 [17].

### 2.2. Effects of Combinations of Vitamin D Derivatives and Carnosic Acid on Cell Differentiation and Cell Cycle Distribution in AML Cells

**Figure 5:**
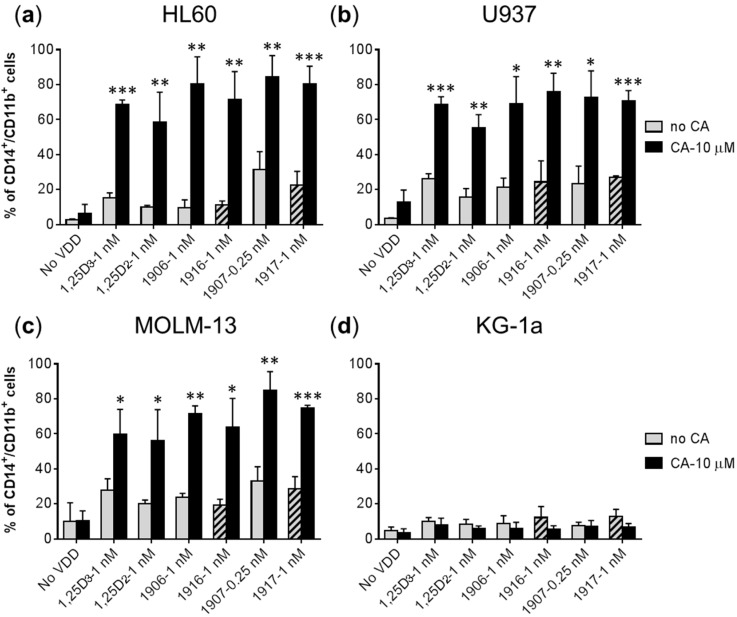
Enhancement of VDD-induced differentiation of AML cells by carnosic acid (CA) is cell type-dependent. Following incubations with the indicated agents for 96 h, the percentage of CD14^+^/CD11b^+^ cells was measured by flow cytometry (**a**–**d**). Hatched columns indicate the effects of the 24*Z* analogs added alone. The data are the means ± SD (*n* = 3). *, *p* < 0.05; **, *p* < 0.01; ***, *p* < 0.001 vs. sum of the effects of single agents.

Irrespective of their different potencies (see above), all the employed VDDs applied at low-nM or sub-nM concentrations were capable of cooperating with the rosemary polyphenol CA to produce enhanced differentiation effects on HL60, U937, and MOLM-13 cells (Figure 5a–c) but not on the most primitive, stem-like KG-1a cells (Figure 5d). Similar to our previous studies (e.g., [17,21,35]), we used a non-cytotoxic concentration of CA (10 μM) that is bioavailable, at least in rodents. For instance, following a single oral administration of 90 mg/kg CA in rats, the plasma level of this polyphenol was maintained at about 30 μM for at least 24 h [36].

**Figure 6:**
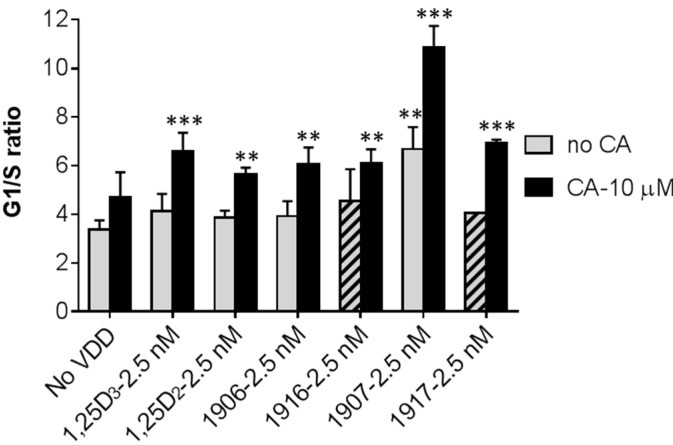
VDDs and CA cooperate in the inhibition of G1-to-S cell cycle transition in MOLM-13 cells. Cells were treated with the indicated concentrations of VDDs and CA, alone and in combination, for 48 h. The cellular DNA content was then determined by propidium iodide staining using flow cytometry. Hatched columns indicate the effects of the 24*Z* analogs added alone. The data are the means ± SD (*n* = 3). **, *p* < 0.01; and ***, *p* < 0.001 vs. untreated control.

In addition to the highest sensitivity of MOLM-13 cells to the prodifferentiation effects of VDDs among the other tested AML cell lines (see Figure 3, Table 1 and [17]), these cells also exhibited the highest susceptibility to the growth-inhibitory effects of PRI-1906, PRI-1907, and their 19-*nor* analogs (PRI-5201 and PRI-5202, respectively) as well as their combinations with CA [17]. Therefore, we examined whether these effects are associated with changes in cell cycle distribution in MOLM-13 cells. The results demonstrated that, among the different VDDs added at a low concentration (2.5 nM), only PRI-1907 significantly elevated the G1/G0 cell population concomitant with a decrease in the percentage of the S phase (data not shown). This resulted in a significant increase in the G1/S ratio, which was further strongly increased in the presence of CA (Figure 6). Notably, while CA and the other VDDs alone did not significantly affect the G1/S ratio, it was significantly elevated by the all the VDD/CA combinations tested (Figure 6).

**Figure 7:**
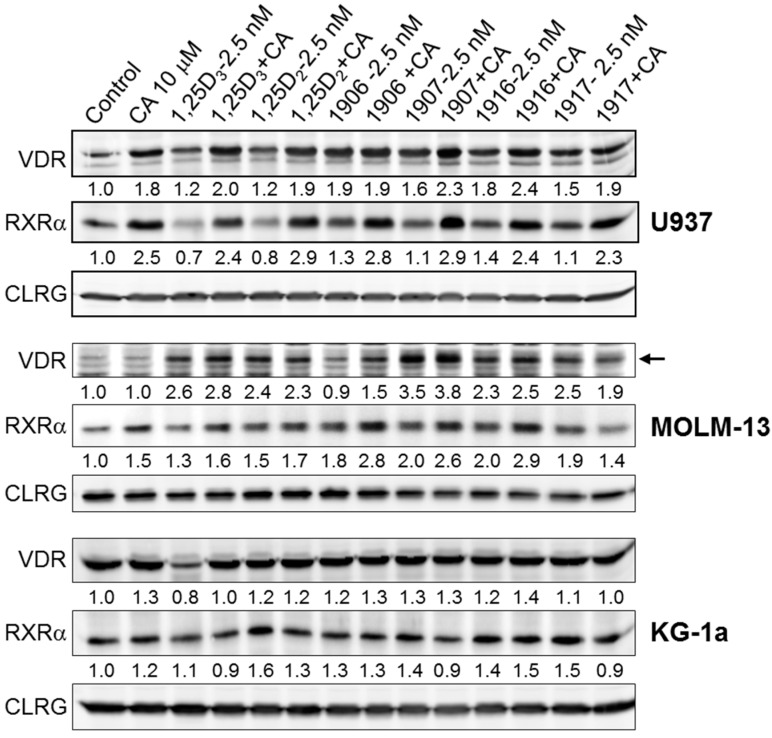
VDDs and CA cooperate in increasing the protein levels of VDR and RXRα in U937 and MOLM-13 cells, but not in KG-1a cells. A Western blot assay was performed following cell treatment with the indicated concentrations of CA and VDDs, alone and in combination, for 48 h. Calregulin (CLRG) was used as the protein loading control. Absorbance values for VDR and RXRα, normalized to those of CLRG, are displayed under each protein band relative to control values. Representatives of 3 similar experiments are shown.

Interestingly, in the cell lines that responded to VDD/CA combinations with enhanced cell differentiation, e.g., in U937 and MOLM-13 cells (Figure 5b,c), treatment with either CA or VDD alone as well as with the combinations resulted in increased expression of the vitamin D receptor (VDR) and its heterodimeric partner, retinoid X receptor (RXRα), as shown in Figure 7. On the other hand, in KG-1a cells that were insensitive to VDD/CA combinations (see Figure 5d), such increases in VDR and RXRα were not evident (Figure 7).

## 3. Discussion

The present study is part of our ongoing research that aims to develop an effective differentiation inducer of the vitamin D_2_ origin with minimal or no calcemic toxicity. We have previously demonstrated that PRI-1906 and PRI-1907, the 1,25D2 analogs with extended and rigidified side-chains, exhibit increased anti-leukemia potency, as compared to 1,25D3 [27,30]. While PRI-1906 is substantially less calcemic than 1,25D3, the much stronger differentiation inducer PRI-1907 is also much more toxic [37,38]. To improve these lead compounds, we recently introduced the 19-*nor* modification of the A-ring of PRI-1906, resulting in PRI-5201 [28]. This double-modified analog exhibited substantially higher differentiation activity in AML cells with only a slightly elevated calcemic effect, as compared to PRI-1906. On the other hand, such modification of PRI-1907 made the resulting PRI-5202 a slightly more potent differentiation inducer and, importantly, greatly reduced its calcemic activity, even below that of 1,25D3 [17,38]. These data support the hypothesis that the anticancer and calcemic activities of VDDs are regulated separately and, perhaps, independently as a result of complex interactions between the A-ring and side-chain structural moieties of the VDDs and ligand binding domain (LBD) of the VDR [39].

Since the above 19-*nor* analogs are still quite calcemic for potential drug candidates, we now explore further structural transformations of our lead vitamin D_2_ compounds. Thus, using a panel of AML cell lines, we characterized the biological effects of the 24*Z* modification of the side-chains of PRI-1906 and PRI-1907 resulting in their geometric analogs, PRI-1916 and PRI-1917, respectively [29]. The principal finding of this study is that, while the 24*Z* modification either had no significant effect or tended to increase the differentiation-inducing potency of PRI-1906, it markedly lowered the potency of PRI-1907 (Table 1). The biochemical basis for such surprisingly different consequences for the two analogs remains to be elucidated. However, practically, this finding suggests that, if employed for further improvement of 1,25D2 analogs, the 24*Z* modification should preferentially be combined with the natural structure of the terminus of the side chain, i.e., C-25 dimethyl, as in PRI-1906, and not C-25 diethyl, as in PRI-1907.

Another important result was that when the two parent vitamin D compounds, 1,25D2 and 1,25D3, were directly compared at the same spectrophotometrically validated concentrations, the prodifferentiation potency of 1,25D2 was found to be significantly lower than that of 1,25D3 in all the AML cell lines tested (Table 1). Interestingly, while the lower differentiation potency of some vitamin D_2_ compounds (e.g., 1,25D2 or PRI-1917), as compared to 1,25D3, correlated with their reduced ability to transactivate DR3-VDREs in U937 cells (Figure 4), a similarly less efficient VDRE activation was observed even for PRI-1907 (Figure 4) or PRI-5202 [17], which acted much stronger than 1,25D3 as differentiation inducers and were capable of upregulating VDR protein levels (Figure 7 and [17]). The nature of these differences remains unclear. One possible scenario is that the differentiation-inducing effects of vitamin D_2_ derivatives may be mediated to a larger extent by non-DR3-type VDREs, e.g., by everted repeat (ER) ER9 [40]. Another possibility is that some nongenomic mechanisms (reviewed in [41]) make a greater contribution to the induction of differentiation by 1,25D2 and its analogs compared with 1,25D3. However, a recent study has shown that 1,25D2 and its derivative paricalcitol were much less effective than 25D3, 1,25D3, and its analog elocalcitol in a rapid stimulation of l-type Ca^2+^ channel-mediated Ca^2+^ influx in human aortic smooth muscle cells [42]. Thus, further mechanistic studies are needed to clarify the mode of action of vitamin D_2_ derivatives in AML cells.

Finally, we show here that, similar to 1,25D3 and irrespective of their relative differentiation-inducing potencies, 1,25D2 and its novel analogs PRI-1916 and PRI-1917 could cooperate at low concentrations with the plant polyphenol CA to markedly potentiate cell differentiation and inhibition of G1-to-S cell cycle transition in a cell type-dependent manner (Figure 5 and Figure 6). The fact that the enhanced differentiation effects of these and the previously described VDD/CA combinations in sensitive AML cell lines were associated with upregulation of both VDR and RXRα protein levels (Figure 7 and [17,35,43]) supports the positive role of the nuclear receptor to vitamin D in the potentiation of VDD-induced differentiation of AML cells by plant polyphenols. Notably, different VDD/CA combinations are not only active against AML cells in culture. We have shown that combined treatment with CA-rich rosemary extract and 1,25D3 analogs results in strong cooperative antileukemic effects in syngeneic mouse AML models in vivo, without inducing hypercalcemia [44,45]. Taken together, the above findings may open up new directions for the development of effective and safe vitamin-D-based combination differentiation therapy of AML.

## 4. Materials and Methods

### 4.1. Chemicals, Antibodies and Plasmids

1,25D3, 1,25D2, and analogs PRI-1906, PRI-1907, PRI-1916 and PRI-1917 were synthesized at the Chemistry Department of the Pharmaceutical Research Institute (PRI; Warsaw, Poland). Carnosic acid (>98%) was obtained from Nanjing Chemlin Chemical Industry Co. (Nanjing, China). Antibodies against VDR (C-20), RXRα (D-20) and calregulin (H-170) were purchased from Santa Cruz Biotechnology (Dallas, TX, USA). The VDRE×6-Luc reporter construct containing a 6-fold direct repeat 3 (DR3) VDRE sequence [34] was kindly provided by Leonard P. Freedman (Memorial Sloan-Kettering Cancer Center, New York, NY, USA). The pGL2-(Spp-1)_2_ luciferase reporter construct containing two copies of the osteopontin (Spp1) DR3-type VDRE (VDRE×2-Luc) [46] was a gift from Roman Pérez Fernández (University of Santiago de Compostela, Spain). Renilla luciferase expression construct (pRL-null vector) was purchased from Promega (Madison, WI, USA) and served as an internal transfection standard. Stock solutions of carnosic acid and VDDs were prepared in absolute ethanol. The exact concentrations of 1,25D3 and 1,25D2 in stock solutions were determined spectrophotometrically in 3-μL quartz capillaries (UltroSpec 2100 Pro, GE Healthcare, Little Chalfont, UK) at 264 nm, applying the extinction coefficients (ε) of 18,300 M^−1^·cm^−1^ and 19,400 M^−1^·cm^−1^, respectively.

### 4.2. Cell Culture

KG-1a cells (ATCC-CCL-246.1) were obtained from Eitan Fibach (Hadassah Medical Center, Jerusalem, Israel). HL60 myeloblastic leukemia cells (ATCC-CCL-240) were obtained from Rachel Levy (Ben-Gurion University of the Negev). U937 promonocytic leukemia cells (ATCC-CRL-1593.2) were purchased from American Type Culture Collection (Rockville, MD, USA). MOLM-13 monocytic leukemia cells (ACC 554) were purchased from Leibniz Institute DSMZ-German Collection of Microorganisms and Cell Cultures (Braunschweig, Germany). Cells were grown in RPMI 1640 medium supplemented with 10% FCS, penicillin (100 U/mL), streptomycin (0.1 mg/mL), and 10 mM HEPES (pH = 7.4) in a humidified atmosphere of 95% air and 5% CO_2_ at 37 °C.

### 4.3. Determination of Cell Differentiation and Cell Cycle Distribution by Flow Cytometry

Cells were seeded at 5 × 10^4^ cells/mL and treated with test agents or vehicle (≤0.2% ethanol) for 96 h. Aliquots of 5 × 10^5^ cells were harvested, washed with PBS and incubated for 45 min at room temperature with 0.3 μL MO1-FITC and 0.3 μL MY4-RD1 (Beckman Coulter) to determine the expression of myeloid surface antigens CD11b and CD14, respectively, as described previously (e.g., [17]). The cell cycle distribution was determined by propidium iodide staining of DNA and analyzed by flow cytometry, as described previously [18].

### 4.4. Preparation of Whole Cell Lysates and Western Blotting

Cells were seeded at 1 × 10^5^ cells/mL and incubated with test agents or vehicle (≤0.2% ethanol) for 48 h. Briefly, cells were lysed in 1% Triton X-100-containing buffer, subjected to SDS-PAGE and immunoblotting as described previously [17]. The VDR and RXRα antibodies were used at 1:200 and 1:1000 dilutions, respectively. Calregulin (1:500) was used as the internal loading control. The protein bands were visualized using the Western Lightning™ Chemiluminescence Reagent Plus (PerkinElmer Life Sciences, Inc., Boston, MA, USA). The absorbance of each band was determined using the Image Quant LAS 4000 system (GE Healthcare Life Science, Little Chalfont, UK).

### 4.5. Transient Transfection and Reporter Gene Assay

U937 cells were transiently co-transfected with the VDRE×6-Luc or VDRE×2-Luc reporter plasmid (0.8 μg) and Renilla luciferase vector (0.2 μg) using JetPEI Reagent (POLYplus-Transfection, Illkirch Cedex, France), as described previously [17]. Transfected cells were exposed to test agents for 24 h followed by measurement of luciferase activity using the Dual Luciferase Reporter Assay system (Promega). The data are presented as the normalized ratios of firefly luciferase to Renilla luciferase activity (relative luminescence units, RLU).

### 4.6. Statistical Analysis

Experiments were repeated at least three times. EC50 values for the differentiation-inducing effects of VDDs were determined by nonlinear regression analysis of dose-response curves (variable slope; three or four parameters). The values are reported as the means ± SD. The significance of the differences between treatments was estimated by unpaired, two-tailed Student’s *t*-test. *p* < 0.05 was considered statistically significant. All statistical analyses were performed with the GraphPad Prism 6.0 program (Graph-Pad Software, San Diego, CA, USA).

## 5. Conclusions

The important structural correlation that we have learned from this study is that the 24*Z* modification of the side chain is probably beneficial for the differentiation activity only in the case of the C-25 dimethyl analogs, such as PRI-1906, and not for the C-25 diethyl analogs (e.g., PRI-1907). Our results also suggest that it may be reasonable to combine the 24*Z* modification with modifications of the A-ring. Comparative evaluation of the calcemic activities of the 24*Z* analogs and next generations of vitamin D_2_ derivatives vs. their differentiation potencies will result in selection of the most suitable candidates for further antileukemic drug development.

## Figures and Tables

**Table 1:** Comparative differentiation-inducing potencies of vitamin D derivatives in acute myeloid leukemia (AML) cells.

Compounds	HL60	U937	MOLM-13	KG-1a
1,25D3	4.58 ± 0.34	1.97 ± 0.19	1.45 ± 0.09	8.65 ± 0.42
1,25D2	8.71 ± 0.62 *	4.31 ± 0.66 *	1.86 ± 0.15	32.29 ± 4.52 **
PRI-1906	4.88 ± 0.02	2.51 ± 0.28	1.67 ± 0.10	4.72 ± 0.97
PRI-1916	3.45 ± 0.07 *	2.87 ± 0.43	1.19 ± 0.08	5.85 ± 1.24
PRI-1907	0.42 ± 0.06	0.56 ± 0.08	0.19 ± 0.02	1.27 ± 0.04
PRI-1917	3.90 ± 0.15 ^##^	2.92 ± 0.51 ^##^	0.57 ± 0.04 ^#^	4.87 ± 0.42 ^#^

EC_50_ values (nM) were calculated by non-linear regression analysis of the dose-response curves for the CD14^+^/CD11b^+^ double-positive cell population (Figure 3). The data are the means ± SD. *, *p* < 0.05 vs. 1,25D3; **, *p* < 0.01 vs. 1,25D3; ^#^, *p* < 0.05 vs. PRI-1907; ^##^, *p* < 0.01 vs. PRI-1907.

## Abbreviations

1,25D2: 1α,25-dihydroxyvitamin D_2_
1,25D3: 1α,25-dihydroxyvitamin D_3_
25D3: 25-hydroxyvitamin D_3_
AML: acute myeloid leukemia
CA: carnosic acid
DR3: direct repeat 3
ER: everted repeat
LBD: ligand binding domain
RXRα: retinoid X receptor
VDDs: vitamin D derivatives
VDR: vitamin D receptor
VDRE: vitamin D response element
